# Strategy to discover full-length amyloid-beta peptide ligands using high-efficiency microarray technology

**DOI:** 10.3762/bjnano.8.243

**Published:** 2017-11-20

**Authors:** Clelia Galati, Natalia Spinella, Lucio Renna, Danilo Milardi, Francesco Attanasio, Michele Francesco Maria Sciacca, Corrado Bongiorno

**Affiliations:** 1 STMicroelectronics, Stradale Primosole, 95121, Catania, Italy; 2 CNR-Istituto di Biostrutture e Bioimmagini, Catania, Italy; 3 IMM-CNR, Catania, Italy

**Keywords:** Alzheimer's disease, florescence, high sensitivity, peptide, silicon oxide, TEM

## Abstract

Although the formation of β-amyloid (Aβ) fibrils in neuronal tissues is a hallmark of Alzheimer disease (AD), small-sized Aβ oligomers rather than mature fibrils have been identified as the most neurotoxic species. Therefore, the design of new inhibitors, able to prevent the aggregation of Aβ, is believed to be a promising therapeutic approach to AD. Unfortunately, the short-lived intermediate structures that occur in a solution along the Aβ aggregation pathway escape conventional experimental investigations and there is urgent need of new tools aimed at the discovery of agents targeting monomeric Aβ and blocking the early steps of amyloid aggregation. Here, we show the combination of high-efficiency slides (HESs) with peptide microarrays as a promising tool for identifying small peptides that bind Aβ monomers. To this aim, HESs with two immobilized reference peptides, (i.e., KLVFF and Semax) with opposite behavior, were investigated for binding to fluorescently labeled Aβ peptide. Transmission electron microscopy was used to demonstrate Aβ fibrillar aggregates missing. The use of HESs was critical to ensure convenient output of the fluorescent microarrays. The resulting sensitivity, as well as the low sample consumption and the high potential for miniaturization, suggests that the proposed combination of peptide microarrays and highly efficient slides would be a very effective technology for molecule profiling in AD drug discovery.

## Introduction

According to the World Health Organization (WHO), the number of people living with Alzheimer’s disease (AD) worldwide is now greater than 47 million, and this number is expected to grow to 75 million by 2030. Alzheimer’s disease leads to nerve-cell death, and scientists believe that the observed build-up of plaque between nerve cells could be the cause of cell death [1]. Two peptides, 40 and 42 amino acids long, known as Aβ40 and Aβ42 amyloid, are the main constituents of the fibrillar plaques [2].

Although amyloid fibrils were initially considered the real causative agents of AD, more recent works have suggested that small-sized Aβ oligomers are the main cytotoxic species [3], and that Aβ monomers may even be neuroprotective [4], in line with these findings, the possibility to detect the small-sized Aβ oligomers was recently demonstrated too [5]. Thus, the design of novel molecules that target Aβ monomers and able to prevent the formation of small toxic oligomers may be considered a promising goal of a number of therapeutic strategies under development. Thus far, a number of different compounds including Congo Red derivatives [6], antibodies [7], osmolytes [8], curcumin [9], carnosine [10], peptide β-sheet breakers [11–13], epigallocatechin-3-gallate (ECGC) [14], D-enantiomeric peptides [15], nonpeptidic β-sheet breakers [16], Sylibins [17], and metal ionophores [18], have been investigated for their potential to treat AD by preventing Aβ aggregation, but none of them could be successfully transferred into clinical practice. It is likely that the limited success of these molecules may be ascribed to our incomplete knowledge of the Aβ aggregation state targeted by the ligand in the solution. In fact, studies addressing the binding of small inhibitors to the Aβ monomers are challenging due to the short lifetimes of the intermediate aggregation states and the highly dynamic nature of their transition into the final fibrils [19–20]. Therefore, there is an urgent need for sensitive tools capable of capturing binding events even at Aβ peptide concentrations at which the Aβ peptide is known to be in a monomeric state.

As a proof of concept for the binding capacity of two peptides with opposite behavior was evaluated in parallel manner; a high-efficiency slide (HES) is used in place of microscope glass slides in order to obtain meaningful results. In the recent past, small molecule microarrays (SMMs) based on isocyanate-modified glass slides have been developed [21]. Several compounds immobilized on the surface were screened for binding to fluorescently-labeled Aβ40. The use of HESs plays a fundamental role because the high sensitivity allows Aβ peptide to be used at very low concentrations. By keeping the Aβ peptide below the critical concentration at which aggregations begin to occur [22–23], the study of the interaction between oligopeptides and Aβ peptide in monomeric form can be possible. In addition, due to the use of HESs, low concentrations of peptide are used achieving a relatively inexpensive assay.

Here, the HES is a silicon-based substrate able to act as a fluorescence amplifier. The intensity of the fluorescence is increased by exploiting the constructive interference phenomena of the electric field of light in the near-surface region [24–25] for a chosen range of frequencies. In addition, a high signal-to-noise ratio due to the low background autofluorescence of the material is obtained [26]. A high purity of the material involved, chemical inertness and stability and atomic flatness of the surface are other important features of the used system [27]. An additional advantage is the ease of implementing on it consolidated coating methods for the hosting of biomolecules.

In this work, for peptide immobilization in a microarray format, we propose one of the most common functionalization methods (an epoxysilane) although different kinds of functionalization processes can be used [28]. To validate the new platform with suitable control experiments, we used two different peptides (i.e., KLVFF and Semax), investigating their ability to bind monomeric Aβ. Residues encompassing the domain 16–20 of the Aβ peptide may act as a self-recognition motif seeding amyloid growth. Therefore, peptides containing this sequence are supposed to interact with Aβ and eventually hinder its self-assembly [29]. This class of peptides was referred to as β-sheet breaker (BSB) peptides. The first example was proposed by Tjernberg, who in the mid-1990’s presented the KLVFF pentapeptide (Aβ16–20) as a strategy to arrest the formation of β-amyloid fibrils, confirming that amino-acid residues located within or close to this region are important for the adoption of the correct β-sheet structure of Aβ [30].

Semax is an hexapeptide (Met–Glu–His–Phe–Pro–Gly–Pro) with a sequence very similar to the ACTH(4–10) fragment (Met–Glu–His–Phe–Arg–Trp–Gly). Differently from the parent molecule ACTH, Semax has no hormonal activity [31]. In vitro and in vivo studies of the peptide activity have shown that Semax affects cognitive brain function with a high efficacy in the treatment of cognitive/memory disorder [32]: nootropic, neurotrophic, neuroprotective and anti-inflammatory effects, a high affinity for copper(II) ions, and a protective ability against metal-induced cell toxicity have been observed [33–36]. Hence, the role of Semax in neurodegenerative disorders can be evaluated.

Prior to assay development, Aβ40 aggregation was evaluated by transmission electron microscopy (TEM) and binding conditions of the peptides under investigation were optimized on the coated silicon oxide surface. Finally, incubation tests between oligopeptides and labeled Aβ40 were carried out.

## Results and Discussion

### Aggregation state of Aβ40 by transmission electron microscopy

**Figure 1:**
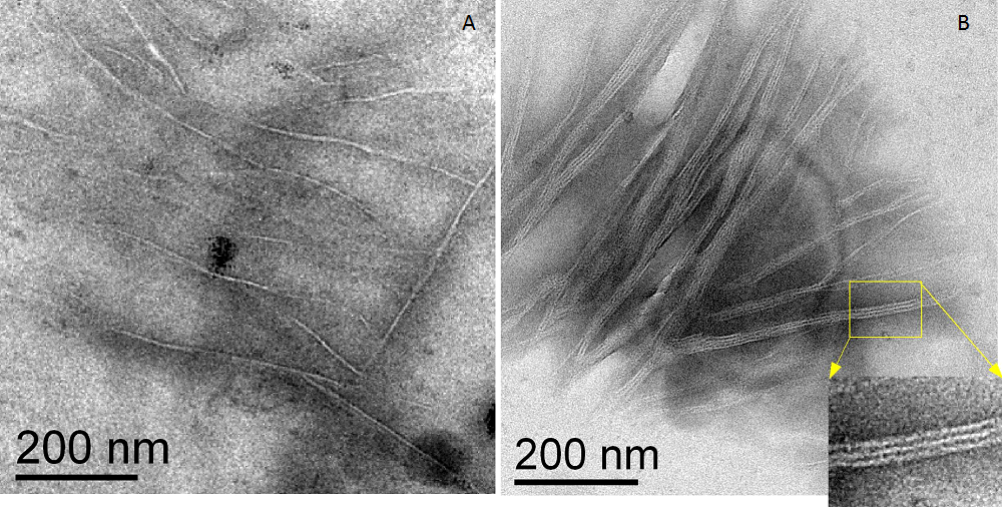
TEM images of Aβ40 fibrils observed after two days of storage. Individual fibril and ribbon-like structures with a branched arrangement are observed in correspondence of a poor (A) and rich fibrillar area (B). The inset shown in (B) is a magnification of a ribbon-like structure consisting of three individual fibrils. The wide of a single filament is around 5 nm.

Though in vitro Aβ40 aggregates slower than the long form Aβ42 [37], aggregations of Aβ40 are formed which needs to be taken into account [38]. In order to rule out the presence of aggregates in the incubation solution, the aggregation state was assessed by TEM. For that purpose, a 1 μM Cy3-Aβ40 solution was placed onto TEM carbon grids and stored in a suitable humidity chamber for different times. Immediately after storage, the grids were then inserted in the TEM vacuum chamber. The high vacuum in the TEM chamber dried them completely and all of the solute species precipitated on the grid carbon layer. After storage in the humidity chamber for one hour, the standard time used for the amyloid incubation in our microarray experiments, no fibrillar structures were observed, in accordance with literature data [21]. The appearance of fibrillar structures was only observed for the longer-aged samples. Figure 1 shows the morphology of fibrils observed after two days of storage in the humidity chamber. The fibrillar structures appear with a diameter of about 5 nm and are morphologically analogue to the branched fibrillar form described elsewhere [39]. Figure 1B displays a more rich area of fibrils, showing the typical 5 nm wide component strands consistent with the ribbon aggregate morphology; inset is the magnified view of the yellow box in Figure 1B in which details of the ribbon aggregates are clearly visible.

### Propensity of the epoxysilane HESs to immobilize peptides

Silanization has been widely-used as a strategy to prepare optimal protein or DNA microarrays [25–26,40], the epoxide groups may bind peptide molecules through the reaction with the basic amino acid residues of the peptide. The propensity of the epoxysilane HESs to immobilize peptides was investigated by measuring the fluorescence of 1 μM Cy3-Aβ40 spot arrays previously assayed until the blocking step. For comparison, reference HESs properly prepared with an O_2_-plasma treatment have been tested. In particular, immediately after the spotting of the amyloid solution, the epoxysilane and the reference slides were inserted into a dark humidity chamber and incubated overnight at 22 °C and 95% relative humidity to immobilize the amyloid spot arrays. The slides were then blocked with BSA buffer following the standard microarray protocol (described in the Experimental section). After BSA blocking, the slides were scanned in order to check the fluorescence of the amyloid spots after the extensive washing involved in the blocking process.

**Figure 2:**
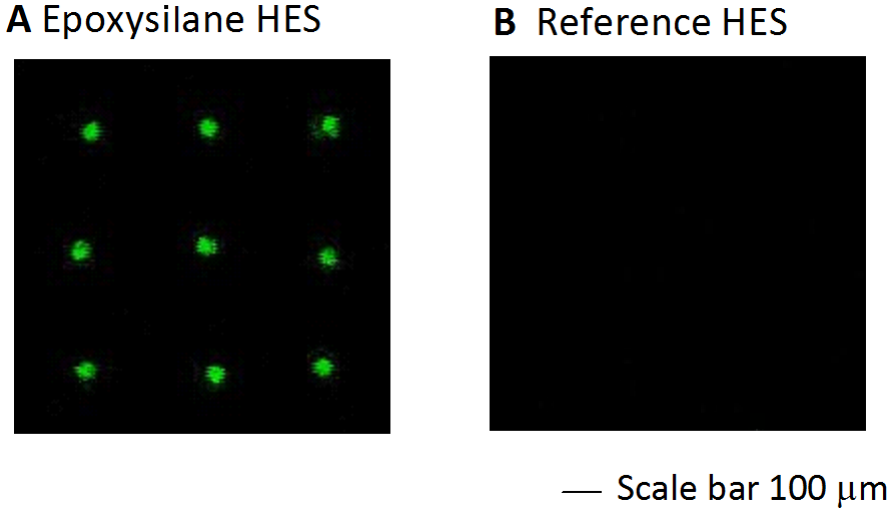
Ability of the epoxysilane-HES to immobilize peptides: fluorescence measurement (60% power laser and gain) are relative to Cy3-labelled Aβ40 peptides spotted on (A) an epoxysilane and (B) on an O_2_-plasma treated HES after the extensive washing involved in the BSA-blocking process.

As shown in Figure 2A, bright amyloid spots are observed on the functionalized slides only. The fluorescence intensity of each spot defines regular circles matched with the spotting scheme. This spot morphology results from the optimal wettability of the epoxysilane slide (see Figure S1 in Supporting Information File 1 for contact angle measurements) that prevents the spreading and coalescence of the spots. As far as reference slide is concerned, in order to minimize effects due to different wettability, stored slide with similar contact angle and very low fluorescence background were used.

The background level of the epoxysilane slide does not essentially increase after the blocking process or differ from the fluorescence background level measured on the reference HESs. This result suggests that the silanization process does not critically modify optical properties of the HESs and the interference phenomena responsible for the fluorescence enhancement may still be preserved upon silanization.

No fluorescence spots have been observed on the reference slide. In this case, the blocking-washing step was effective to fully remove the full-length amyloid spots. After the O_2_-plasma treatment, the surface of the un-epoxysilane slides may likely be rich in Si–OH groups that can be reactive against several environmental species, e.g., water; the silanization process may instead generate a certain number of reactive epoxide groups, randomly oriented at the silicon surface, which may react with the basic amino groups of the full-length amyloid peptide and therefore immobilize the peptide at the silicon surface. The same behavior can be assumed for shorter peptides.

### Test of amyloid-beta 1–40 binding by peptide ligands on HES

The synthesized pentapeptide KLVFF and a commercial therapeutic heptapeptide, Semax, have been assayed to investigate their ability to bind full-length Aβ40 peptides. KLVFF was chosen as reference for its ability to bind to specific regions of the full-length Aβ peptide. This peptide, known as β-sheet breaker, has been specifically designed to inhibit the formation of fibrils; the sequence KLVFF, corresponding to the Aβ 16–20, was identified to bind full-length Aβ and to prevent its fibrillation [30]. Semax was chosen for its different behavior (as will be discussed below).

**Figure 3:**
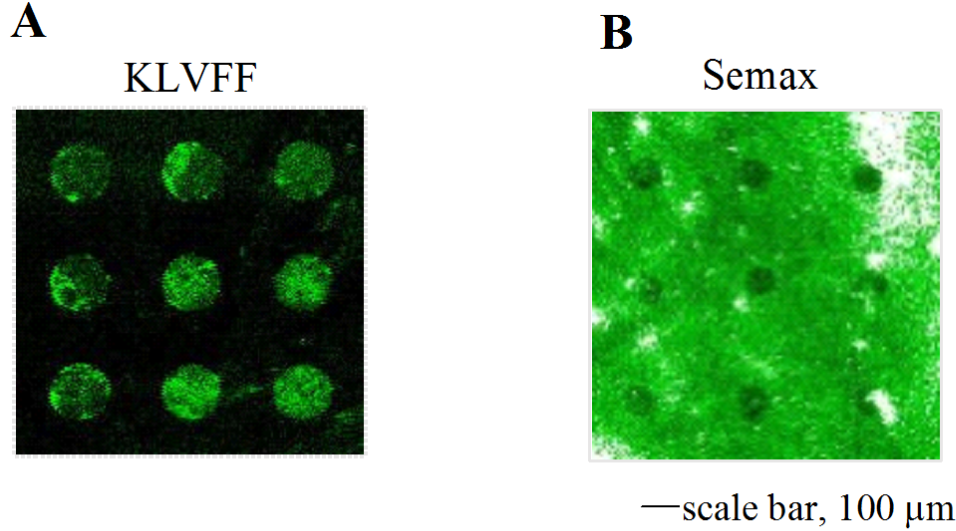
(A) KLVFF and (B) Semax spot arrays on an epoxysilane-coated HES after amyloid incubation. The fluorescence measurements were performed by operating at an optimized condition for laser power and gain (60% laser power and gain for KLVFF and 100% laser power and gain for Semax).

The peptides were tested, as amyloid ligands, using the Cy3-labeled Aβ40 conjugate for fluorescence detection. As expected, after the immobilization of the unlabeled peptides only very weak signals (Figure S2A, Supporting Information File 1) were detected, deriving from the light scattering of salts contained in the phosphate buffer used to prepare the peptide spotting solution, and no bright spots were detected after the blocking step (Figure S2B, Supporting Information File 1). These fluorescence measurements suggest that the washing operations were effective to remove the buffer salts from the epoxysilane surface. However, because of the expected behavior similar to amyloid peptides (as shown in Figure 2), the unlabeled peptides were not removed. Figure 3 reports the incubation data. The fluorescent images clearly show the different propensity of the peptides under investigation to bind to the full-length Aβ40. Bright Cy3-Aβ40 spots were detected in the KLVFF spot array (Figure 3A) and black spots were measured on Semax (Figure 3B). In order to emphasize the different behavior of the two peptides, the florescence images were acquired with different laser and gain conditions. For incubated KLVFF spots, a lower laser power than for Semax was used. Otherwise, saturation of the detector would have been reached, which is not useful for quantitative assessments.

**Figure 4:**
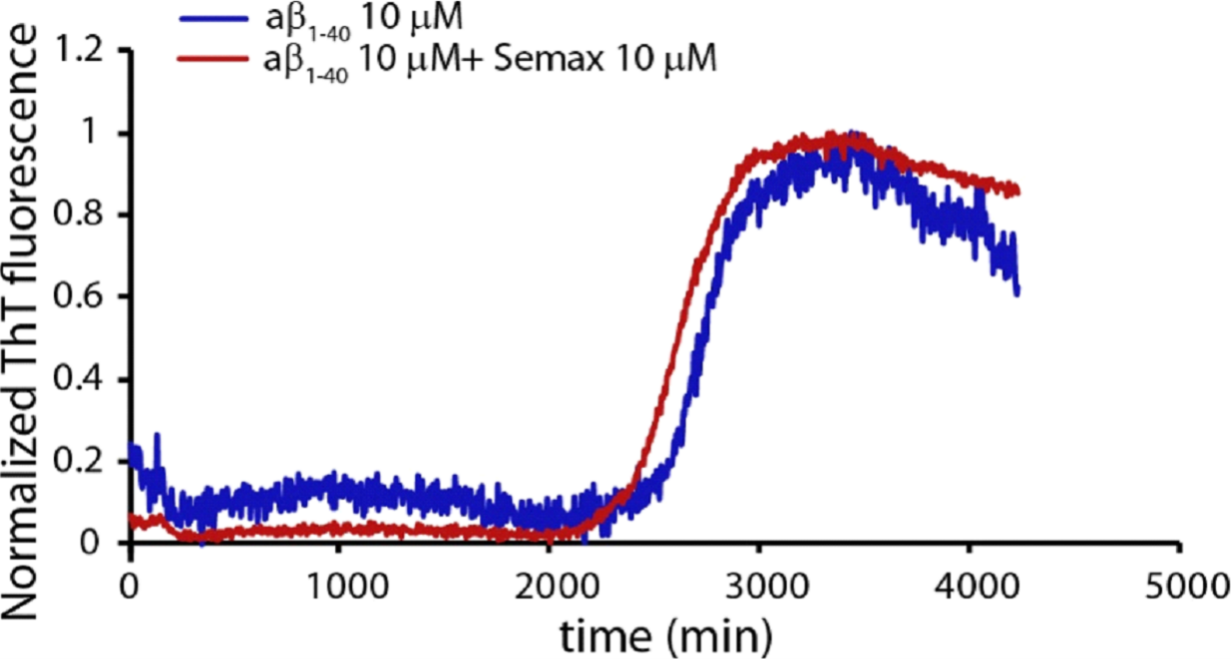
Amyloid fibril growth detected by Th-T fluorescence as a function of the time; 10 µM Aβ40 (blue curve), Aβ40 in presence of equimolar amount of Semax (red curve).

In accordance with the previous work [24], the positive binding interaction between the KLVFF spots and the Cy3-Aβ40 solution occurs, giving rise to the appearance of bright amyloid spots for the KLVFF spot array. Conversely, for the Semax peptide, the formation of black spots , was observed. This is due to the “blocking effect” phenomenom, likely deriving from an anti-bonding interaction between Semax spots and the Aβ40. In this case, the amyloid preferentially interacts with the blocked epoxysilane surface rather than binding to the Semax spots. This suggests a poor ability of Semax to interact with the Aβ peptide and is consistent with the absence of any anti-aggregating activity of Semax monitored by Th-T fluorescence assays (Figure 4).

Finally, to assess the sensitivity of the method, a quantitative evaluation was carried out on the coated HES. The experimental conditions of the assay are detailed in Supporting Information File 1. Briefly, solutions of known concentrations ranging from 0.01 to 1 μM of Cy3-Aβ40 were dispensed on the functionalized HSE (Figure S3, Supporting Information File 1). The calibration curve of the fluorescence intensity as a function of the amyloid concentration is reported in Figure S4 (Supporting Information File 1). A value of ca. 10^3^ amyloid molecules captured per square micrometer was achieved. In order to find potential improvements of method, and to determine the limits of detection (LOD), a scanarray acquisition of the calibration slide was acquired at high laser power and high photomultiplier gain (Figure S4A, Supporting Information File 1). Under these conditions the highest spotted concentrations are fully saturated. At a concentration of 0.01 μM slight saturation occurs, allowing to evaluate the LOD by measuring signal and background over 16 spots and evaluating the signal corresponding to three times the average standard deviation of the background. The results show an improvements of the LOD of more than two orders of magnitude. The obtained results support the employment of HES as effective method for improving the sensitivity of microarrays (small molecule, peptide, and antibody) for multiplex detection. In this study, the HES platform has exhibited a high usefulness for studying the behavior of peptides aimed to identify potential therapeutics for Alzheimer’s disease.

## Conclusion

Binding assays have been performed in a microarray format by using highly efficient slides. This highly sensitive optic system has important features: very high purity of the material involved, chemical inertness and stability, and atomic flatness of the surface. Additional advantages of this substrate are the easy implementation of consolidated coating methods for the hosting of biomolecules and its simple structure which allows a fabrication with high reproducibility.

In particular, the ability of two different peptides to bind full-length Aβ peptides has simultaneously been studied, using concentrations in a range in which the Aβ peptide is known to be in a monomeric state. Expected results were obtained indicating that the explored platform can be very useful in the rational drug design for AD.

In the near future, as the effect of environmental factors, such as the presence of small molecules and metal ions, and related methods such as static or in-flow incubations, play a significant role, we shall evaluate these effects and in parallel manner. A novel microfluidic screening system including the high efficiency substrates [41] for very sensitive analyses with low consumption of reagents and short measuring times is under investigation in our laboratory.

## Experimental

### Chemicals

Phosphate-buffered saline, hydrogen peroxide (29%), ammonium hydroxide (25%), hydrochloric acid (37%), methanol, dimethyl sulfoxide (DMSO), anhydrous toluene and 3-glycidyloxypropyltrimethoxysilane (GOPs) were acquired from Sigma-Aldrich. Bovine serum albumin (BSA), IgG-free and protease-free was acquired from Jackson ImmunoResearch Laboratories (West Grove, PA, USA). Cyanine3-labeled human amyloid-beta 1–40 (Cy3-Aβ40), was acquired from Phoenix Pharmaceuticals.

### Preparation of peptides

The KLVFF peptide was kindly gifted by Dr. G. Pappalardo. As described in [42] this peptide was assembled using the microwave-assisted solid-phase peptide synthesis strategy on a Liberty Peptide Synthesiser. All Fmoc-amino acids were introduced according to the TBTU/HOBT/DIEA activation method, as reported elsewhere [11]. Semax was purchased from Caslo, with a purity of 98%. To remove any pre-existing aggregated form of Aβ40, samples were subjected to a disaggregating procedure before carrying out each experiment. The following protocol was applied: The samples were dissolved in 1,1,1,3,3,3-hexafluoro-2-propanol (HFIP) at a concentration of 1 mg/mL and incubated at 37 °C for 1 h. HFIP was removed by gentle streaming of argon, the peptide film was dissolved again in 1 mL HFIP, loaded on the plate and frozen at −30 °C for 5 h, then lyophilized overnight. The lyophilized samples on the plate were then dissolved in 10 mM phosphate buffer pH 7.2 to a concentration of 10 µM.

### Fabrication of HESs

**Figure 5:**
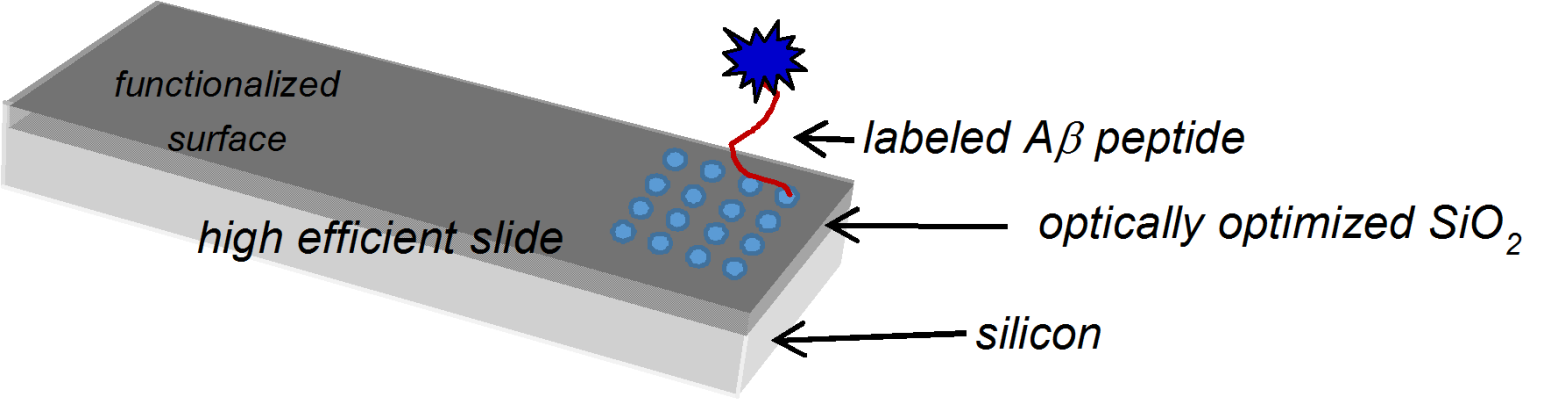
HES with a sketch of incubation test site. The peptide array is represented by blue dots.

HESs were prepared by exploiting the highly controlled materials and technologies conventionally used for the construction of microelectronics devices. The detection sensitivity is essentially determined by the signal-to-noise (S/N) ratio. A simple approach to increasing the S/N ratio involves the use of low-fluorescence materials as substrates. High-purity substrates may be obtained only by starting from reagents with high purity under highly controlled reaction conditions. This is the rule of silicon technology, in which(100) single-crystal 99.999999999% pure silicon is used as the starting material. After standard cleaning, etching in diluted HF (aq), and rinsing in deionized water, single crystalline, Czochralski grown, silicon(100) slices were mounted in a conventional furnace operating at atmospheric pressure. Thermal oxidation was performed at a controlled rate in order to reproducibly grow layers of silicon oxide of a defined thickness. Thermal growth allows high-quality SiO_2_ films to be obtained with very low auto-fluorescence and very low contamination. The wafers were then cut in pieces to the exact dimensions of conventional microscope slides. Figure 5 shows a highly schematic functionalized high efficiency slide including a sketch of the site of incubation between the spotted peptides and labeled Aβ40.

### Coating of HESs with GOPs

HESs were pretreated with an oxidant solution (H_2_O/NH_4_OH/H_2_O_2_, 5:1:1) for 1 min at 80 °C, rinsed with deionized water, immersed in a methanolic solution (CH_3_OH/HCl, 4:1) for 10 min at room temperature, rinsed with water, and dried under a nitrogen flow. Next, the slides were immersed in a solution of GOPs, 1:10 in anhydrous toluene, for 4 h at room temperature. The silanization process was performed in a glove box (MB10 Compact, Braun) that maintained a nitrogen atmosphere with levels of less than 1 ppm of oxygen and humidity. Epoxysilane slides were rinsed and dried in a vacuum chamber and stored under vacuum conditions until the microarray experiments.

Water wettability of the functionalized surface was evaluated by contact angle measurements. Analysis was conducted on the dried slide immediately after the silanization process using a CAM KSV instrument operating in static mode and located in a class-1000 clean room. Drops of Millipore water were dispensed with a volume of 1 μL on at least five representative areas of the epoxysilane slide. The contact angle was then evaluated using the KSV controller instrument software and a medium value of (50 ± 1)° was obtained (Figure S1, Supporting Information File 1).

To test the propensity of the silanizated surface to immobilize peptides, reference silicon slides were properly prepared. Oxygen-plasma treatment was utilized to remove surface contaminations. This cleaning processing is useful to obtain slides with low fluorescence background, becoming a mandatory path when no wet cleaning protocol can be applied, such as in innovative integrated technologies [41,43].

The O_2_-plasma cleaning was performed using the plasma etching system, Sentech 591, using 60 sccm of oxygen fluxing for 2 min. The resulting epoxysilane coating provide a proper surface for the immobilization of peptides in a microarray format. Moreover, due to the low fluorescence background, which does not significantly differ from the background level of the reference slide (i.e., the O_2_-plasma treated silicon slide), they are suitable to be used as substrates for fluorescence microarray assays.

### Aggregation states of the Aβ40 by TEM

Aliquots of 3 μL of the 1 μM Cy3-Aβ40 solution were dispensed onto carbon-coated copper grids for electron microscopy (Cy3-Aβ40 conjugate was dissolved in a saline phosphate buffer containing 10 vol % of DMSO, 0.01 M Phosphate, 0.154 M sodium chloride, pH 7.4). To replicate the experimental conditions involved in a typical peptide microarray assay performed in this work, electron microscopy grids after amyloid dispensing were stored for at least 1 h, the time used for the incubation of the peptide spot arrays with the Cy3-Aβ40 solution, as well as for longer times (up to 48 h). To avoid the drying of the amyloid solution dispensed on the grids, they have been stored in a humidity chamber that was provided with a constant-humidity environment at a controlled temperature (22 °C and 95% relative humidity, RH). The physical state of the amyloid peptides was then tested by TEM. TEM analysis was performed with a Jeol JEM 2010 electron microscope operating at 200 kV accelerating voltage.

### Preparation of peptide microarray

KLVFF and Semax peptides were dissolved in phosphate buffer at a concentration of 1 mg/mL and aliquots were stored at −80 °C until use. Cy3-Aβ40 conjugate was dissolved in a saline phosphate buffer containing 10 vol % of DMSO (0.01 M Phosphate, 0.154 M sodium chloride, pH 7.4) at a concentration of 1 μM in accordance with the producer specification and stored at −80 °C until use.

KLVFF, Semax peptide solutions and Cy3-Aβ40 were patterned on epoxysilane HESs using a PerkinElmer Piezorray non-contact microarraying system. The volume of the spotted drops was 333 pL ± 5%. After dispensing, all slides were placed in a humidity chamber at 22 °C and 95% RH overnight. Spotted slides were then scanned with a PerkinElmer Scanarray express laser scanner apparatus. The slides were then blocked with 1% (w/v) BSA in phosphate buffer for 1 h, washed three times under stirring with phosphate buffer for 2 min, and finally washed with water for 10 min. For the Aβ40 incubation test, the blocked slides were incubated with the 1 μM Cy3-Aβ40 solution in a humidity chamber at 22 °C and 95% RH. After 1 h, the incubation was stopped with the phosphate washing and the final water washing. The dried slides were finally scanned for the fluorescence measurements.

### Th-T fluorescence measurements

The fluorescence emission spectra of Th-T undergoes a red shift upon incorporation into β-sheet amyloid structures. An aggregation assay was performed on a VarioSkan Flash from Thermo Scientific fluorescence 96-well plate reader, programmed to agitate the plate for 30 s before measuring fluorescence emission. Readings were taken every 10 min over a range of 4200 min. To minimize evaporation effects, the wells were sealed with a transparent heat-resistant plastic film. Fluorescence excitation was at 440 nm and emission detected at 480 nm. To minimize errors during sample preparation, we freeze-dried the 10 µM Aβ40 solutions directly in the well plate. The Th-T experiments were carried out at pH 7.2, at 37 °C and in 10 mM phosphate buffer and replicated three times. The Semax concentration was 10 µM. The Th-T concentration was 20 μM.

## Supporting Information

Supporting Information includes additional data relative to the characterization of material surface, quantitative evaluations, and a study of a possible interaction between the anchored-amyloid and amyloid-target.

File 1 Additional figures, quantitative evaluations and further experimental data.

## References

[1] Hardy J (2002). Neurobiol Aging.

[2] Kang J, Lemaire H-G, Unterbeck A, Salbaum J M, Masters C L, Grzeschik K-H, Multhaup G, Beyreuther K, Müller-Hill B (1987). Nature.

[3] Haass C, Selkoe D J (2007). Nat Rev Mol Cell Biol.

[4] Giuffrida M L, Caraci F, Pignataro B, Cataldo S, De Bona P, Bruno V, Molinaro G, Pappalardo G, Messina A, Palmigiano A (2009). J Neurosci.

[5] Wennmalm S, Chmyrov V, Widengren J, Tjernberg L (2015). Anal Chem.

[6] Frid P, Anisimov S V, Popovic N (2007). Brain Res Rev.

[7] Solomon B (2007). Curr Opin Invest Drugs (BioMed Cent).

[8] Yang D-S, Yip C M, Huang T H J, Chakrabartty A, Fraser P E (1999). J Biol Chem.

[9] Begum A N, Jones M R, Lim G P, Morihara T, Kim P, Heath D D, Rock C L, Pruitt M A, Yang F, Hudspeth B (2008). J Pharmacol Exp Ther.

[10] Attanasio F, Convertino M, Magno A, Caflisch A, Corazza A, Haridas H, Esposito G, Cataldo S, Pignataro B, Milardi D (2013). ChemBioChem.

[11] De Bona P, Giuffrida M L, Caraci F, Copani A, Pignataro B, Attanasio F, Cataldo S, Pappalardo G, Rizzarelli E (2009). J Pept Sci.

[12] Soto C, Sigurdsson E M, Morelli L, Kumar R A, Castaño E M, Frangione B (1998). Nat Med.

[13] Soto P, Griffin M A, Shea J-E (2007). Biophys J.

[14] Bieschke J, Russ J, Friedrich R P, Ehrnhoefer D E, Wobst H, Neugebauer K, Wanker E E (2010). Proc Natl Acad Sci U S A.

[15] Brener O, Dunkelmann T, Gremer L, van Groen T, Mirecka E A, Kadish I, Willuweit A, Kutzsche J, Jürgens D, Rudolph S (2015). Sci Rep.

[16] Rzepecki P, Nagel-Steger L, Feuerstein S, Linne U, Molt O, Zadmard R, Aschermann K, Wehner M, Schrader T, Riesner D (2004). J Biol Chem.

[17] Sciacca M F M, Romanucci V, Zarrelli A, Monaco I, Lolicato F, Spinella N, Galati C, Grasso G, D’Urso L, Romeo M (2017). ACS Chem Neurosci.

[18] Grasso G, Santoro A M, Lanza V, Sbardella D, Tundo G R, Ciaccio C, Marini S, Coletta M, Milardi D (2017). Coord Chem Rev.

[19] Pannuzzo M, Milardi D, Raudino A, Karttunen M, La Rosa C (2013). Phys Chem Chem Phys.

[20] Pannuzzo M, Raudino A, Milardi D, La Rosa C, Karttunen M (2013). Sci Rep.

[21] Chen J, Armstrong A H, Koehler A N, Hecht M H (2010). J Am Chem Soc.

[22] Harper J D, Lansbury P T (1997). Annu Rev Biochem.

[23] O’Nuallain B, Shivaprasad S, Kheterpal I, Wetzel R (2005). Biochemistry.

[24] Volle J-N, Chambon G, Sayah A, Reymond C, Fasel N, Gijs M A M (2003). Biosens Bioelectron.

[25] Bras M, Dugas V, Bessueille F, Cloarec J P, Martin J R, Cabrera M, Chauvet J P, Souteyrand E, Garrigues M (2004). Biosens Bioelectron.

[26] Arrabito G, Galati C, Castellano S, Pignataro B (2013). Lab Chip.

[27] Cretich M, di Carlo G, Longhi R, Gotti C, Spinella N, Coffa S, Galati C, Renna L, Chiari M (2009). Anal Chem.

[28] Cretich M, Galati C, Renna L, Condorelli G G, Gagni P, Chiari M (2014). Sens Actuators, B.

[29] Chalifour R J, McLaughlin R W, Lavoie L, Morissette C, Tremblay N, Boulé M, Sarazin P, Stéa D, Lacombe D, Tremblay P (2003). J Biol Chem.

[30] Tjernberg L O, Näslund J, Lindqvist F, Johansson J, Karlström A R, Thyberg J, Terenius L, Nordstedt C (1996). J Biol Chem.

[31] Rzepecki P, Steger L N, Feuerstein S, Linne U, Molt O, Zadmard R, Aschermann K, Wehner M, Schrader T, Riesner D (2006). J Neurochem.

[32] Ashmarin I P, Nezavibatko V N, Myasoedov N F, Kamenskii A A, Grivennikov I A, Ponomareva-Stepnaya M A, Andreeva L A, Kaplan A Y, Kosheleva V B, Ryasina T V (1997). Zh Vyssh Nervn Deyat im I P Pavlova.

[33] Bashkatova V G, Koshelev V B, Fadyukova O E, Alexeev A A, Vanin A F, Rayevsky K S, Ashmarin I P, Armstrong D M (2001). Brain Res.

[34] Potaman V N, Antonova L V, Dubynin V A, Zaitzev D A, Kamensky A A, Myasoedov N F, Nezavibatko V N (1991). Neurosci Lett.

[35] Storozhevykh T P, Tukhbatova G R, Senilova Ya E, Pinelis V G, Andreeva L A, Myasoyedov N F (2007). Bull Exp Biol Med.

[36] Tabbì G, Magrì A, Giuffrida A, Lanza V, Pappalardo G, Naletova I, Nicoletti V G, Attanasio F, Rizzarelli E (2015). J Inorg Biochem.

[37] Seilheimer B, Bohrmann B, Bondolfi L, Müller F, Stüber D, Döbeli H (1997). J Struct Biol.

[38] Hansson O, Zetterberg H, Peder B P, Andreasson U, Londos E, Minthon L, Blennow K (2007). Dementia Geriatr Cognit Disord.

[39] Goldsbury C S, Wirtz S, Müller S A, Sunderji S, Wicki P, Aebi U, Frey P (2000). J Struct Biol.

[40] Morales-Narváez E, Montón H, Fomicheva A, Merkoçi A (2012). Anal Chem.

[41] Renna L, Galati C, Spinella N M R, Coffa S (2011). Optically Accessible Microfluidic Diagnostic Device. U.S. Patent.

[42] Sinopoli A, Magrì A, Milardi D, Pappalardo M, Pucci P, Flagiello A, Titman J J, Nicoletti V G, Caruso G, Pappalardo G (2014). Metallomics.

[43] Renna L, Galati C, Spinella N, Mazzillo M, Abbisso S, Fallica P G (2015). Sens Actuators, B.

