# Supplementary material for: Strategy to discover full-length amyloid-beta peptide ligands using high-efficiency microarray technology
Source: Beilstein J Nanotechnol. 2017 Nov 20;8:2446–53. doi: 10.3762/bjnano.8.243 (PMC5704750; doi:10.3762/bjnano.8.243)
Supplement: File 1 — Additional figures, quantitative evaluations and further experimental data. [file Beilstein_J_Nanotechnol-08-2446-s001.pdf]

Supporting Information

for

Strategy to discover full-length amyloid-beta peptide ligands using high-efficiency microarray technology

Clelia Galati*[1], Natalia Spinella[1], Lucio Renna[1], Danilo Milardi[2], Francesco Attanasio[2], Michele Francesco Maria Sciacca[2] and Corrado Bongiorno[3]

Address: [1]STMicroelectronics, Stradale Primosole, 95121, Catania, Italy; [2]CNR-Istituto di Biostrutture e Bioimmagini, Catania, Italy and [3]IMM-CNR, Catania, Italy

Email: Clelia Galati* - clelia.galati@st.com

* Corresponding author

Additional figures, quantitative evaluations and further experimental data

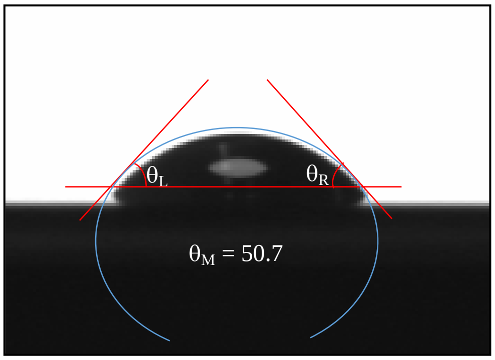

Figure S1: Water contact angle of epoxysilane HES. The value of the water contact angle, θ_M , is calculated from the mean value of the left and right angle, θ_L and θ_R respectively, formed between the drop contour line (blue line) and the projection of the slide surface (red line). The drop volume is 1 μL.

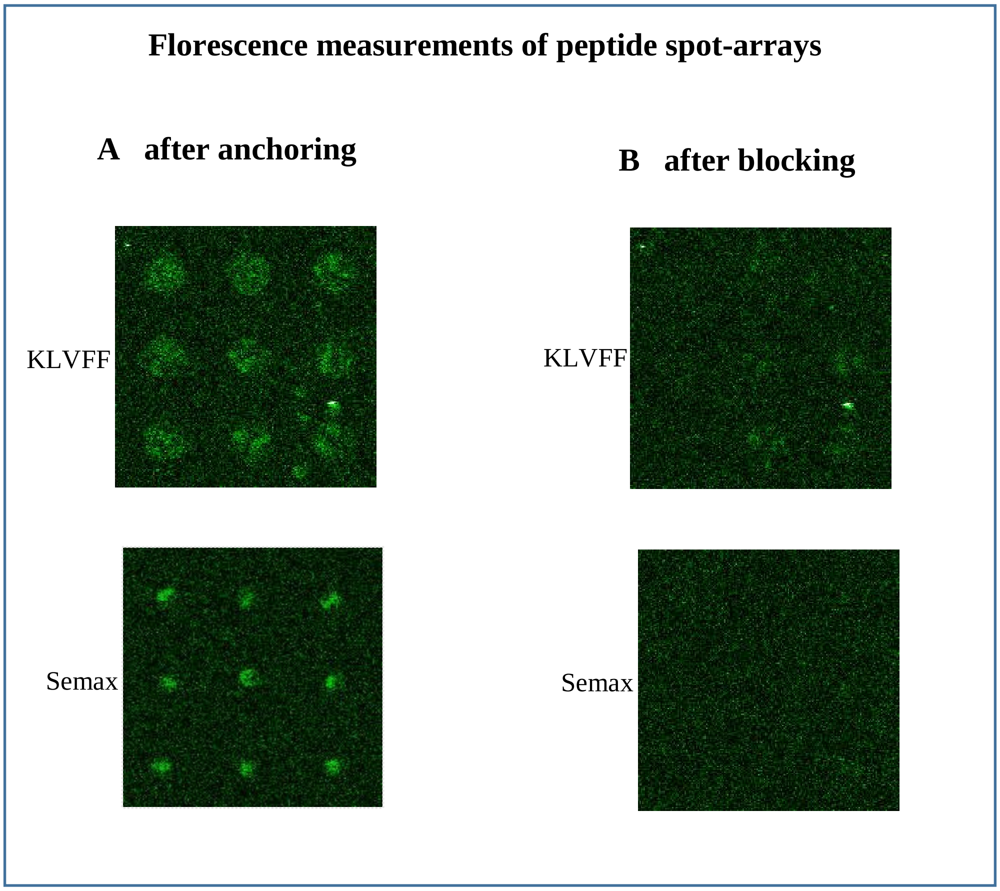

Figure S2: Fluorescence measurements on HES after peptide anchoring (A) and blocking procedures (B). To obtain brilliant spots, images were acquired under sensor saturation conditions for laser power and gain, 100%.

Quantitative evaluation

To evaluate the sensibility of the amyloid incubation assay the fluorescence of Cy3-Aβ40 spot arrays was calibrated analyzing three Cy3-labeled amyloid solutions spotted on epoxysilane-coated HESs at different amyloid concentrations. For sample preparation, a proper amount of the lyophilized Cy3-Aβ40 product has been dissolved in a phosphate-buffered saline containing dimethyl sulfoxide (0.01 M phosphate, 0.154 M sodium chloride, pH 7.4, 10 vol % of DMSO). The PBS was added until the amyloid concentration reached a final value of 1 μM. Diluted amyloid solutions were prepared by adding the PBS to fresh aliquots of the 1μM amyloid solution in order to reach 1:10 and 1:100 dilutions. After preparation amyloid solutions were stored at −80 °C until use.

Three sets of 4 × 4 spot arrays, replicated two-times, have been spotted for each of the amyloid solutions by using a PerkinElmer Piezorray non-contact system. The volume of spotted drops was 333 pL ± 5%. A reference solution, consist of dilution buffer was spotted at the same time to assure a negative control.

Florescence was measured on the spotted HES with a PerkinElmer Scanarray instrument immediately after the amyloid-spotting procedure. Various acquisition conditions were tested in order to get all spots visible although below the detector saturation value (Figure S4B). Under sensor saturation conditions (100% power laser and gain) all the amyloid concentrations clearly appear as white spots (Figure S4A). In addition, PBS spots are also detected. As discussed in the full paper, this signal derive from secondary luminescence effects due to the salt components.

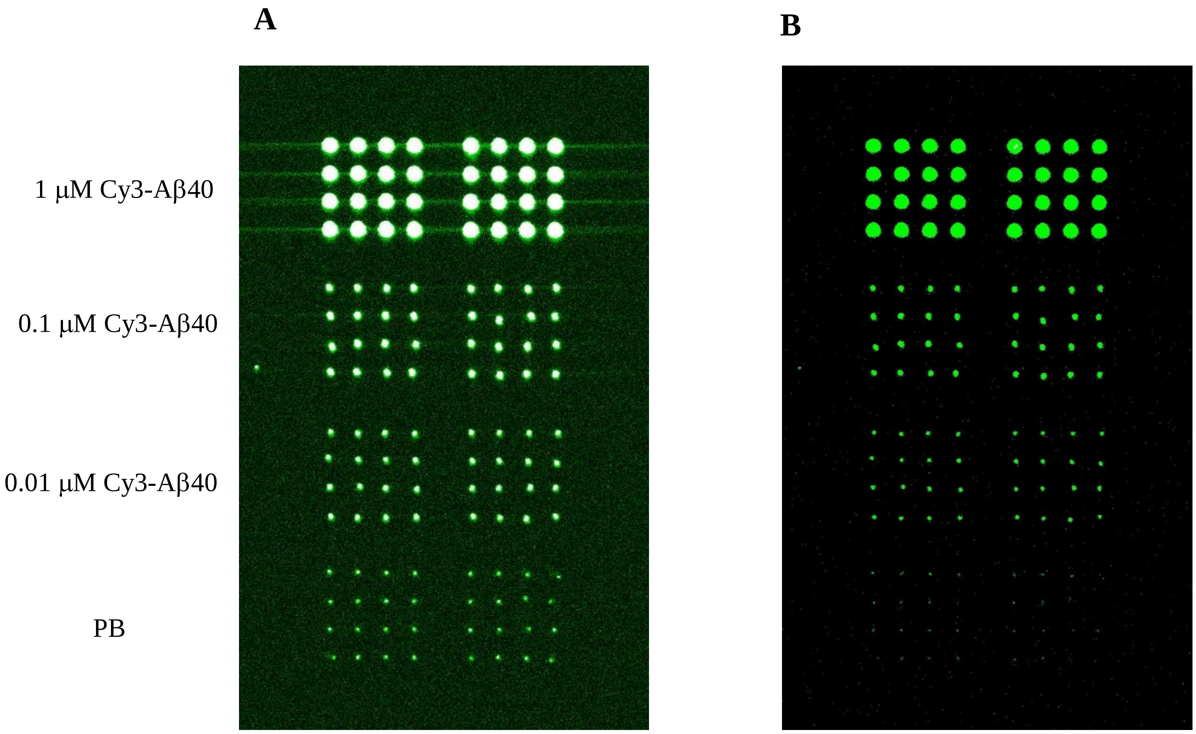

Figure S3: Fluorescence measurements of Cy3-labeled amyloid and phosphate-buffer saline solutions arrayed on HESs. The images are acquired in saturated and optimized conditions, A (100% laser power and gain) and B (60% laser power and gain).

Spots and background intensity have been evaluated and corresponding averaged (over 16 spots) net signal has been plotted (Figure S4 - data relative to the measurement acquired at 60% of laser power and gain). The fitted raw data have been used to extrapolate number of bonded molecules after incubation step. Error bars determined as standard deviation of 16 spots measured intensity has been evaluated.

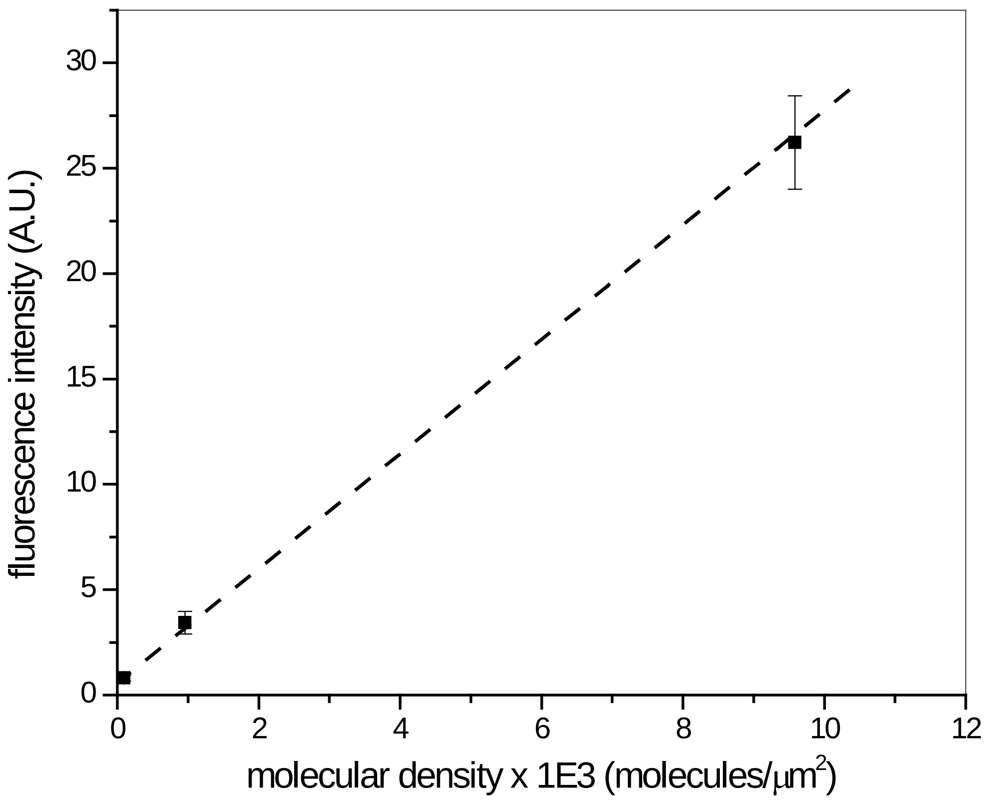

Figure S4: Intensity and linear fit of fluorescent molecules spotted at various concentrations. Error bars determined as standard deviation of 16 spots measured intensity.

Study of any interactions between the anchored amyloid and amyloid target

To explore the tendency of full-length amyloid peptides to form molecular aggregates at the HESs surface through amyloid-amyloid molecular interactions, the binding capacity of Aβ40 spots was compared with the binding capacity of the KLVFF amyloid sequence. Cy3-labeled Aβ40 and KLVFF peptides were arrayed on an epoxysilane-coated slide from a 1 μM and 1 mg/mL peptide solution, respectively. Cy3-Aβ40 incubation test has been performed by assaying, at the same time, the KLVFF sequence and amyloid beta peptide, with a 1 μM Cy3-labeled Aβ40 solution and the relative fluorescence was measured on the dried slide before and immediately after the amyloid incubation.

The amyloid incubation is detailed in the Experimental section of the main manuscript. Briefly, after the spotting step, peptide and amyloid spot arrays were anchored in a humid chamber and

then BSA-blocked. After N_2-drying, the BSA-blocked slide was scanned in order to check for peptide and amyloid spots still visible despite the extensive washings involving in the procedure. Finally, fluorescence measurements have been performed after the amyloid incubation to check for fluorescent spots deriving from the binding interaction with the KLVFF and Cy3-Aβ40 spot arrays.

As shown in Figure S5A, BSA-blocked HESs does not show fluorescence for the KLVFF spot array. This is consistent with the absence of a fluorescent cyanine marker for this peptide. Fluorescent spots have been instead detected for the Cy3-Aβ40 spot array.

However, after amyloid incubation among the Cy3-Aβ40 and KLVFF spot arrays, the last become clearly visible thanks to the appearance of brilliant Cy3-Aβ40 spots. In contrast, Cy3-Aβ40 spots retain the majority of their morphology and brilliance and no fluorescent enhancement is observed after the amyloid incubation (Figure S5 B).

These data suggest that when a KLVFF peptide is anchored at a solid support, it may link a full-length amyloid beta peptide only if the peptide sequence is well-exposed at the solid–liquid interface where the interaction with the solution occurs, just like in the case of KLVFF peptides spotted on the HESs surface. When the KLVFF sequence is instead embedded into a full-length Aβ40 sequence, its ligand propensity is reduced due to the occurring of steric effects that inhibit the interaction. This hypothesis matches with our incubation data, in fact we observed a positive variation of the fluorescence intensity only when the KLVFF sequence is anchored at the support surface, but no variation of the fluorescence signal takes place when it is a part of a full-length amyloid molecule. As a result no formation of amyloid aggregates can occurs on HESs surface, under the experimental conditions used in this work

A BSA-blocked HESs

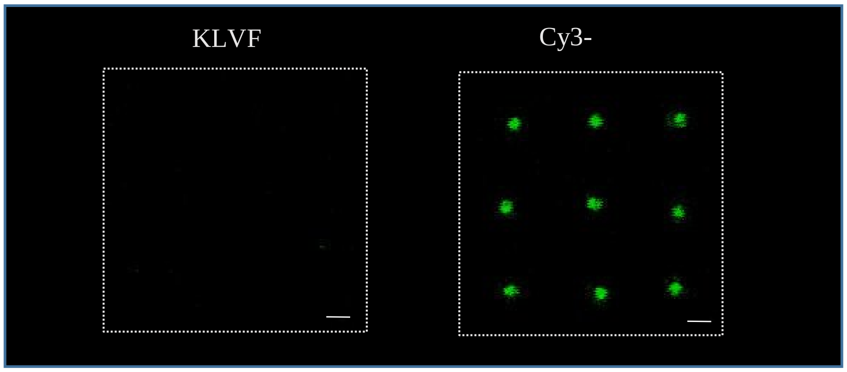

B Cy3-Aβ40 incubated

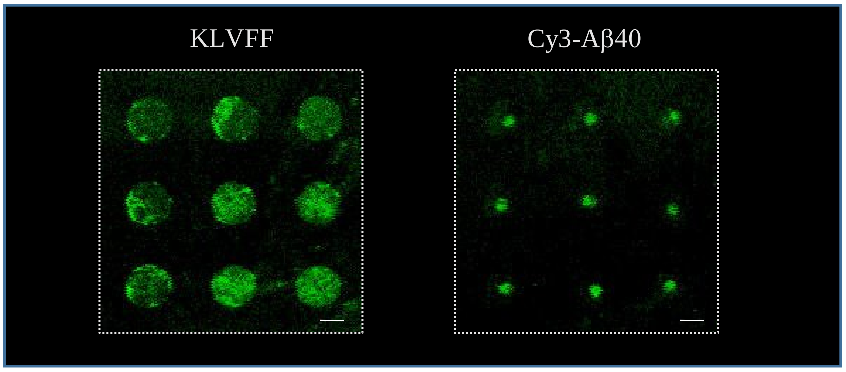

Figure S5: Fluorescence measurements of KLVFF and Cy3-Aβ40 spot arrays before (A) and after incubation with 1 μM Cy3-Aβ40 solution (B). Scale bar, 100 μm
